# Sandwich-Structured Carbon Nanotube Composite Films for Multifunctional Sensing and Electrothermal Application

**DOI:** 10.3390/polym16172496

**Published:** 2024-09-01

**Authors:** Canyi Lu, Encheng Liu, Qi Sun, Yiqin Shao

**Affiliations:** 1 College of Textile Science and Engineering (International Institute of Silk), Zhejiang Sci-Tech University, Hangzhou 310018, China; lucanyi2027@163.com (C.L.); 2021327100079@mails.zstu.edu.cn (E.L.); 2 School of Mechanical Engineering, Zhejiang Sci-Tech University, Hangzhou 310018, China; 2021330301022@mails.zstu.edu.cn; 3 Engineering Research Center of Technical Textiles, Ministry of Education, Donghua University, Shanghai 201620, China

**Keywords:** carbon nanotube film, PDMS, sensor, multifunctional composite, joule heating

## Abstract

Electro-conductive films with excellent flexibility and thermal behavior have great potential in the fields of wearable electronics, artificial muscle, and soft robotics. Herein, we report a super-elastic and electro-conductive composite film with a sandwich structure. The composite film was constructed by spraying Polyvinyl alcohol (PVA) polymers onto a buckled conductive carbon nanotube-polydimethylsiloxane (CNTs-PDMS) composite film. In this system, the PVA and PDMS provide water sensing and stretchability, while the coiled CNT film offers sufficient conductivity. Notably, the composite film possesses high stretchability (205%), exceptional compression sensing ability, humility sensing ability, and remarkable electrical stability under various deformations. The produced CNT composite film exhibited deformation (bending/twisting) and high electro-heating performance (108 °C) at a low driving voltage of 2 V. The developed CNT composite film, together with its exceptional sensing and electrothermal performance, provides the material with promising prospects for practical applications in wearable electronics.

## 1. Introduction

Flexible sensors constitute a fundamental component within the domain of flexible electronic devices [1,2,3]. Current scholarly endeavors focus on the exploration of novel materials [4] characterized by enhanced flexibility, heightened sensitivity [5,6], and reduced cost [7]. Concurrently, efforts are directed towards the development of multifunctional composites possessing attributes of flexibility, stretchability [8], and conductivity [9,10]. Flexible electronic devices can be applied in fields such as wearable technologies [4,5], smart textiles [6], and electronic skins [7,8], promising enhanced user comfort and convenience. The development of these technologies requires systematic research in device design [9], material selection [10], and manufacturing processes [11]. Flexible sensors encompass a broad range of research areas, such as wearable health monitoring devices [12,13]. Due to their pliable and stretchable characteristics, flexible sensors can better conform to the human body surface, providing high-precision physiological parameter monitoring [14]. They enable real-time monitoring of human movement and health status, supporting personalized health management through data utilization. Additionally, certain implantable sensors can achieve real-time monitoring of physiological parameters within the body, providing essential support for medical diagnosis and treatment. This is significant for early disease detection and personalized therapy [15]. Flexible electrothermal materials exhibit the capacity to promptly generate heat when electrically activated, while maintaining the flexibility and bendability of the material. These materials primarily consist of conductive materials and matrix materials. The conductive materials, which are the main components that conduct current and provide heating, can be classified into metallic and non-metallic materials. Metallic materials include metal fibers and metal nanowires, while non-metallic materials encompass carbon fibers, carbon nanomaterials, and conductive polymers. The matrix material is crucial for determining the flexibility, stability, and thermal consistency of the flexible electrothermal materials [16]. Flexible electrothermal materials possess a wide range of applications, including smart clothing [17], medical care [18,19], and building heating [20]. Through the integration of these materials, automatic heating functionality can be achieved in garments, providing warmth to the wearer in cold environments. Additionally, the excellent properties of flexible electrothermal materials make them ideal heating elements for thermal therapy and physiotherapy devices in the medical field [21]. They provide a robust foundation for the development of new intelligent healthcare devices, driving the healthcare sector towards heightened levels of intelligence and personalization.

Clemens et al. developed a pressure-sensitive textile material based on flexible plastic optical fiber technology. When the pressure is applied to these fibers in the textile, the cross-section will change reversibly, and the change of the transmitted light intensity will be detected at the same time to measure the pressure change. The flexible sensor can be integrated into textiles, providing promising technology and application prospects in the field of smart textiles [22]. Du et al. developed a highly sensitive hydrogel-based flexible sensor through ultrafast polymerization utilizing a tannic acid (TA)-Fe^3+^ dynamic redox system, resulting in excellent adhesion performance and remarkable stretchability. The sensor demonstrated a low detection limit, high sensitivity at small strains, fast response and recovery times, and reliable applications in accurate human motion monitoring and handwriting recognition, opening new possibilities for wearable electronic devices, electronic skins, and human–computer interaction applications [23]. Bu et al., employing the finite element method (FEM), examined factors affecting the performance of flexible capacitive pressure sensors utilizing a porous elastomeric polymer as the dielectric layer. The findings reveal that both structural and material attributes of the porous polymer impact sensor performance [24]. In Li’s research, in summary, a photocurable resin/carbon nanotube nanocomposite was fabricated using aligned CNTs in an acrylic matrix, displaying a rapid increase in conductivity followed by stabilization beyond the percolation threshold. Leveraging this nanocomposite, a touch-based human interface device (HID) was created using a DLP 3D printer, enabling the cost-effective design of sensors with diverse styles and shapes, without the need for complex circuitry [25].

Currently, more research in the field of flexible strain sensors focuses on the utilization of CNTs. Bae et al. prepared flexible PDMS/CNT patches and introduced β-cyclodextrin as a sensing medium. They found that signal intensity depends on the composite’s electrical conductivity, highlighting the need for further study [26]. Due to their conductivity, when CNTs are combined with other flexible materials, these composites exhibit a noticeable dependence on mechanical strain [27,28,29]. When the composite is subjected to tensile force, graphene layers slip and create microcracks. These microcracks reduce the contact area of graphene sheets, leading to an increase in the composite’s resistance [30]. When CNTs are combined with water-soluble materials, the resistance of the original humidity-sensitive material remains nearly unchanged at low relative humidity (RH). However, as the RH increases above 75%, the resistance increases sharply, demonstrating good humidity-switching characteristics [31]. By varying the concentration of multi-walled carbon nanotubes (MWCNTs), different nanocomposite strain gauges can be prepared and embedded in epoxy resin. Since mechanical strain affects the resonant frequency of MWCNTs-epoxy resin strain gauges, these nanocomposite films can be used for the wireless detection of mechanical strain, enabling the preparation of wireless strain sensors [32].

Many scholars, both domestically and internationally, are conducting research on flexible electrothermal materials. Xie et al. developed a PU/PPy composite film using a wet process and in situ polymerization method. Experimental tests revealed that this composite film exhibited excellent heating performance and stability, capable of heating to 108 °C within 28 s, indicating its broad application prospects for wearable devices [33]. Mohamed et al. prepared a CNTF carbon nanofilm using the FCCVD method. Their research found that CNTF can reach a steady-state temperature of 310 °C at a low voltage of 2.5 V, with uniform temperature distribution, suggesting significant potential for applications in multifunctional structures and wearable electronics [34]. Yan et al. prepared an MWCNTs-PDMS composite using effective solution casting and curing techniques. Experiments demonstrated that this composite film has an excellent heating–cooling cycle performance and good thermomechanical stability, making it a high-performance flexible electrothermal element widely applicable in floor heating, medical devices, and functional textiles [35].

Wang et al. developed stretchable, multidirectional strain sensors by embedding carbon nanotube layers into PDMS using a digitally controlled printer. They demonstrated tunable gauge factors and high durability, comparing the sensor data to a motion capture system, with less than 20% deviation, and fabricated rosette-type sensors for multi-axis strain measurement [36]. Chen et al. fabricated a stretchable and transparent strain sensor using a spray deposition and transfer method to create a PDMS/CNTs/PDMS composite with an ultrathin conductive layer. The sensor showed high stretchability, optical transparency, and reliable performance in detecting both subtle and large strains [37]. Compared to traditional PDMS/CNT strain sensors, the new approach enhances performance and expands applications by incorporating a tunable sensitivity printing layer. This innovation allows for high-sensitivity, multidirectional strain detection, addressing current limitations and advancing flexible and wearable electronics.

Herein, we demonstrated an innovative sandwich-like super-elastic composite film with high sensing and electrical-thermal performance by spraying the water-sensitive PVA polymers onto a highly elastic and conductive CNTs-PDMS composite film. The thin CNT film, manufactured by floating catalyst chemical vapor deposition with excellent electrical conductivity and thermal conductivity, was adopted as a conductive layer. The PDMS with good elasticity was selected as the polymer layer. The surface and cross-section morphology of the composite CNT films were identified by using SEM. The mechanical performance of the PDMS-CNTs-PVA composite films were evaluated via tensile testing under cyclic loading. The electrical properties under tensile and compression were characterized, and their simulation models were also established. Furthermore, various body movements were selected to investigate the sensor performance to confirm the potentiality of prepared devices for practical applications. The electrical heating responses and efficiency, after applying various applied cyclic voltages, were examined. The electrical and thermal performance proved the potential application of the multifunctional film.

## 2. Experimental Materials and Methods 

### 2.1. Materials

The continuous CNT film was provided by the Suzhou Institute of Nano-Tech and Nano-Bionics, Chinese Academy of Sciences. The CNT film was spun directly using aerogel spinning methods by chemical vapor deposition. The pure CNT films were of an average 3 µm thickness, the macroscopic CNT film with randomly oriented CNT bundles had a density of 780 mg/cm^3^. The conductivity of the CNT film was 357 S/cm. PVA, obtained from the Sinopec Shanghai Petrochemical Company, had a degree of polymerization of 1750 and an alcoholysis degree of 98%. PDMS had a specific gravity of 1.07 g/cc and viscosity of 3000 cps (Smooth-on, Inc., Macungie, PA, USA).

### 2.2. Preparation of PDMS-CNTs-PVA Films

A stretchable PDMS substrate was prepared by mixing a silicone elastomer base with a curing agent, and the mixture was placed in a petri dish. The thickness of the PDMS was controlled to be 2 mm. It was then sealed and left to stand in an oven at 45 °C for 10 min. Due to its chemical structure, PDMS is a long-chain polymer with a Si-O-Si backbone. This long-chain structure facilitates molecular entanglement and sliding, giving PDMS its macroscopic viscosity. Additionally, the methyl groups (-CH_3_) on the molecular chains can generate van der Waals forces with adjacent molecules. The cumulative van der Waals forces between the chain segments create strong intermolecular interactions, thereby increasing the adhesion between PDMS and CNTs. The high specific surface area of PDMS further enhances these intermolecular forces, resulting in excellent adhesive properties and flexibility.

Once the silicone rubber solidified at room temperature, the cured PDMS was cut into appropriately sized strips, which were stretched to a certain extent at a specific ratio. The CNT film was subsequently attached to the PDMS substrate, and then allowed the initial composite to elastically recover. PDMS substrates pre-strained to 100% were used for the buckled composite films, with the pre-strain considered approximately equal to the actual strain.

A PVA solution was prepared by adding an appropriate amount of PVA to a mixture of distilled water (80 vol%) and alcohol (20 vol%), and stirring with a magnetic stirrer at 90 °C for 4 h. The PVA was then sprayed evenly on the surface of the composite film and kept in a wet state for 2 min. After spraying, the composite film was placed in an oven at 45 °C to remove excess moisture and dispersant. 

**Figure 1:**
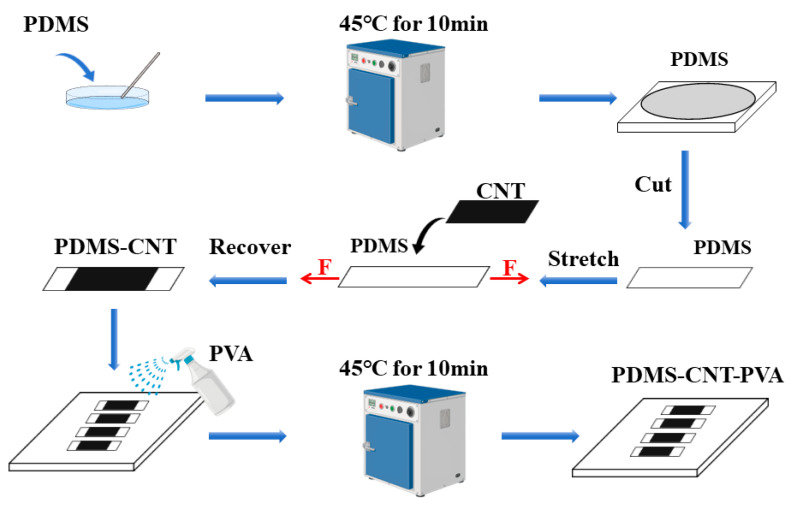
Experimental illustration of PDMS-CNTs-PVA composite film.

This process resulted in the final PDMS-CNTs-PVA composite film. A schematic illustration of the fabrication process is summarized in Figure 1. For cyclic loading films, a length (1 cm) of the composite film was straightly clamped in the middle of a piece of cardboard using silver paste, with copper wires as leads to connect the multi-meter.

### 2.3. Characterization

The tensile behavior and cyclic loading of films were measured by a displacement-controlled tensile tester (XQ-2, Shanghai Xusai Instrument Co., Shanghai, China), with an extension rate of 1 mm/min. Meanwhile, a multi-meter (Agilent 34465A, Keysight, Santa Rosa, CA, USA) was connected to measure the electrical resistance. The surface morphology of the composites was observed using scanning electron microscopy (SEM, Hitachi TM3000, and 5 kV) and Field Emission SEM (FESEM, Hitachi S4800, and 5 kV) to evaluate the CNTs’ structure and the uniformity of the PVA coating. The dynamic resistance changes under compressive stress, in both dry and wet states, were tested using Keysight BenchVue. The electrothermal properties and cyclic loading experiments were carried out using an adjustable constant DC power (LW-K3010D, LONGWEI, Dongguan, Guangdong, China), the voltage was set at between 1 and 5 V, and the temperature changes were observed and recorded by an infrared thermal camera (E85, FTIR, Arlington, VA, USA). To enhance the accuracy of the experiment, more than 20 specimens were tested for each film, and the resulting data were averaged.

## 3. Results and Discussions

### 3.1. Morphologies

**Figure 2:**
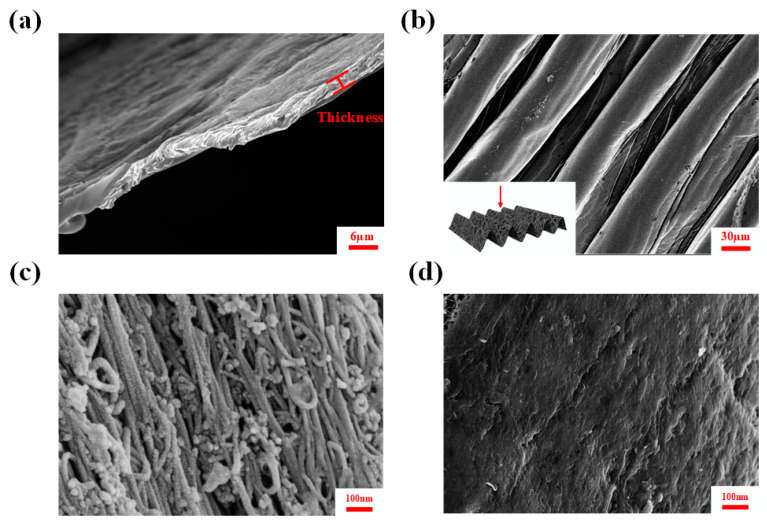
(**a**) Cross-section of CNT film; (**b**) SEM images of buckled PDMS-CNTs-PVA composite film; (**c**) SEM image of CNT bundles; (**d**) PVA polymers on CNT bundles.

As illustrated in Figure 2, the morphologies of PDMS-CNTs-PVA composite films were studied using Scanning Electron Microscopy (SEM). Figure 2a shows the ~3μm thickness of CNT films. The SEM images of buckled PDMS-CNTs-PVA composites are shown in Figure 2b. The CNT film consists of numerous carbon nanotube bundles, as shown in Figure 2c, which depicts the morphology of these bundles before the PVA was applied. After the PVA was sprayed onto the CNT film, significant changes in the material’s surface were observed (Figure 2d). At this stage, the PVA predominantly covered the surface of the CNT film, with minimal polymer infiltration.

### 3.2. Electrical Behavior and Sensing Properties of PDMS-CNTs-PVA Composite Films 

**Figure 3:**
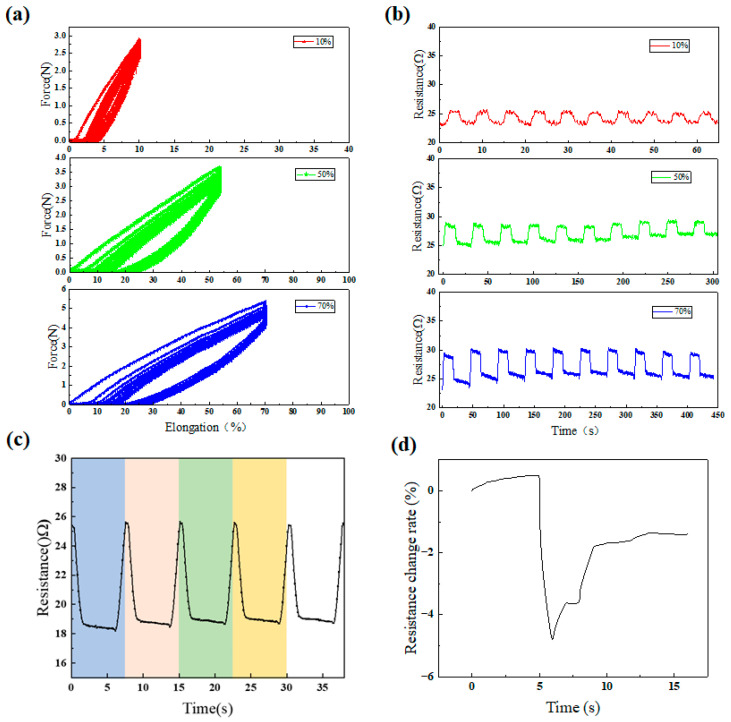
(**a**) Tensile force-elongation curve of PDMS-CNTs-PVA films under cyclic loading; (**b**) electrical resistance changes of the PDMS-CNTs-PVA film during 10 cyclic loading processes with 10%, 50%, and 70% loading strains; (**c**) electrical resistance changes of PDMS-CNTs-PVA films under compression; (**d**) resistance changes of moisture film.

In order to gauge the elasticity strain limit of the CNT composite films, a group of cyclic loading experiments were performed. During the cyclic loading experiments, films were tested at a speed of 1 cm/min, with a stable strain (10%, 50%, and 70%, respectively) for 10 loading cycles. A clear hysteresis loop may be observed in the plot. For elastic strain cyclic loading, the area (strain energy lost) maintained nearly the same level, as shown in Figure 3a,; this process could be attributed to the slippage and fracture of threads, and the reversible deformation of bundles and PDMS.

Figure 3b shows the resistance response over time for the cyclic loading test procedure. It was evident that in each subsequent cycle under different strains, there was a larger resistance change upon reloading, and a permanent resistance change in the unloading state after damage was caused. The composite films demonstrated good stability and sensing performance after the long-term cyclic tensile test. The resistance of the PDMS-CNTs-PVA-coated films remained relatively constant during the subsequent cyclic loading.

**Figure 4:**
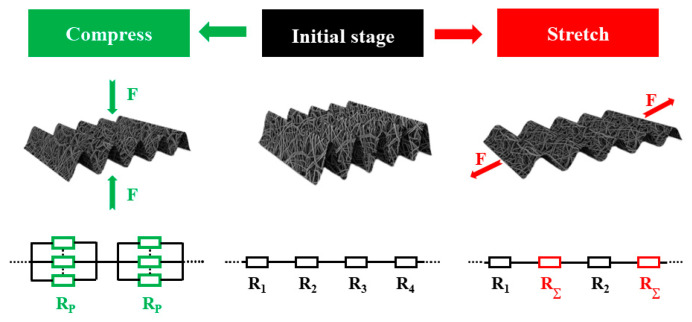
The schematic of the PDMS-CNTs-PVA sensor structure and relative resistance change under stretching and compression.

When pressure was applied to the composite, the buckling state changed, causing parts of the composite to overlap and contact each other. This increased the contact points between CNT bundles and decreased the resistance. When the external force was removed from the PDMS-CNTs composite films, the material returned to its initial state, the contact points between the CNTs decreased, and the resistance increased (Figure 3c). The principle of resistance is illustrated in Figure 4.

### 3.3. Water-Sensing Properties

**Figure 5:**
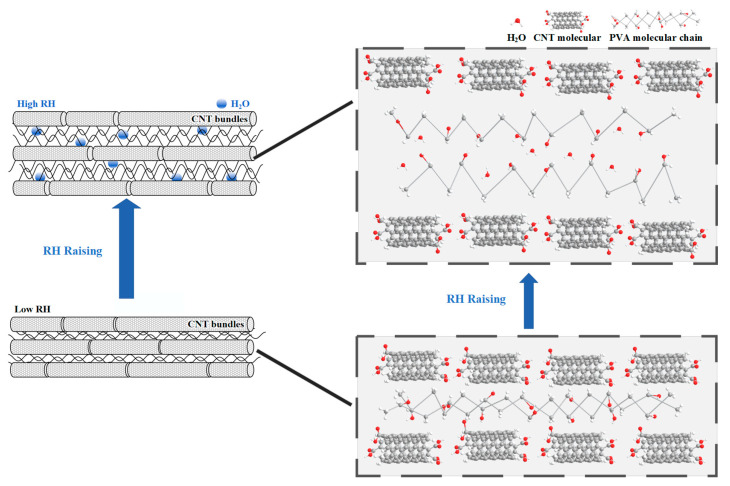
Moisture absorption simulation of PDMS-CNTs-PVA.

To explore the water-sensing behavior of PDMS-CNTs-PVA composite films, the electrical resistance values were measured (Figure 3d). When the composite films were placed in a high-moisture environment, the PVA matrix expands with increasing moisture, resulting in an increase in electrical resistance. Moisture absorption at 100% RH caused the PVA polymer to swell rapidly, leading to a significant increase in the volume of the CNTs-PVA coating and a rapid decrease in the contact points between CNTs, causing a sharp rise in resistance. Subsequently, due to the physicochemical adsorption of water molecules, H^+^ ions were donated to the surface of the CNTs, which decreased the resistance until it stabilized [38,39] (Figure 5).

Water influences the electrical properties of composite films while having negligible effects on their mechanical properties and other performance aspects. Notably, changes in moisture can significantly alter the electrical behavior of these materials, affecting parameters such as resistivity, conductivity, and dielectric constant. However, the mechanical properties, including strength, modulus, and toughness, remain largely unaffected by changes in moisture. These mechanical properties are primarily determined by the intrinsic characteristics of the matrix and reinforcement materials, which exhibit stability under varying moisture conditions.

**Figure 6:**
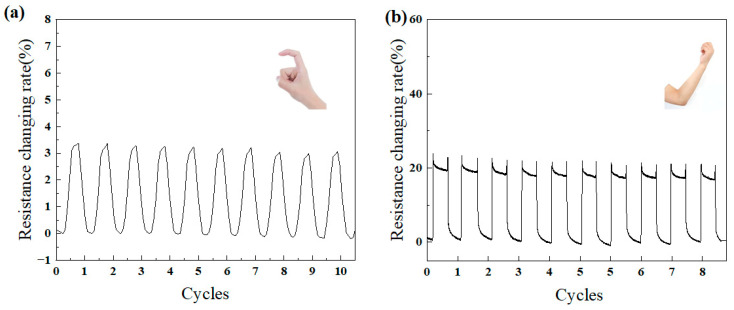
Relative resistance change with different human motions: (**a**) finger, (**b**) elbow.

The capability of the PDMS-CNTs-PVA composite films for detecting human body movements was investigated. To assess its feasibility in dynamic conditions, the sensor was applied to joints such as the elbow and fingers. As shown in Figure 6, when the elbow is bent at a 90° angle and held for 20 s, or when the finger is bent to 90°, the electrical properties of the composite film undergo significant changes, as reflected in the variation of resistance. The PDMS-CNTs-PVA composite films demonstrated sufficient sensitivity for detecting body movements across diverse scenarios, rendering them highly suitable for applications in health monitoring.

### 3.4. Electrothermal Performance

**Figure 7:**
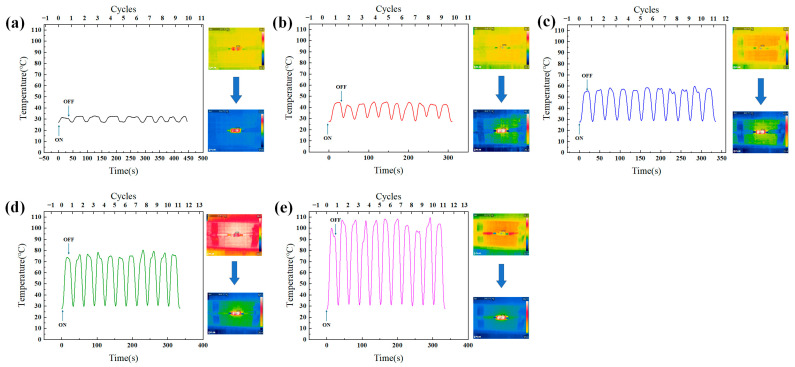
Electrothermal temperature variations of the CNT films. The power supplies for (**a**) 1 V, 0.05 A; (**b**) 2 V, 0.1 A; (**c**) 3 V, 0.15 A; (**d**) 4 V, 0.2 A; (**e**) 5 V, 0.25 A.

The steady-state temperatures of the CNT films (2 × 4 cm^2^) under various applied voltages were monitored and depicted in Figure 7. The CNT films rapidly reached the maximum temperature, and reached the steady-state equilibrium temperature in about 20 s, which exhibited a high thermal conversion and short-time response.

The temperature reached a maximum steady state when the heat generated by the electrical power equaled the heat radiated plus the heat dissipated. Once the temperature stabilized, the external power was removed, and the temperature of the CNT films dropped to room temperature. As the applied voltage increased, the temperature of the CNT films exhibited a simultaneous gradient increase. The temperatures of the CNT films reached 32.5, 44.8, 55.8, 75.6, and 106.7 °C under applied voltages of 1, 2, 3, 4, and 5 V, respectively (Figure 7), demonstrating the activation of Joule heating performance, even at a low voltage of 1 V. The low trigger voltage and high steady-state temperature were attributed to its excellent electrical conductivity (~35.7 S/cm).

**Figure 8:**
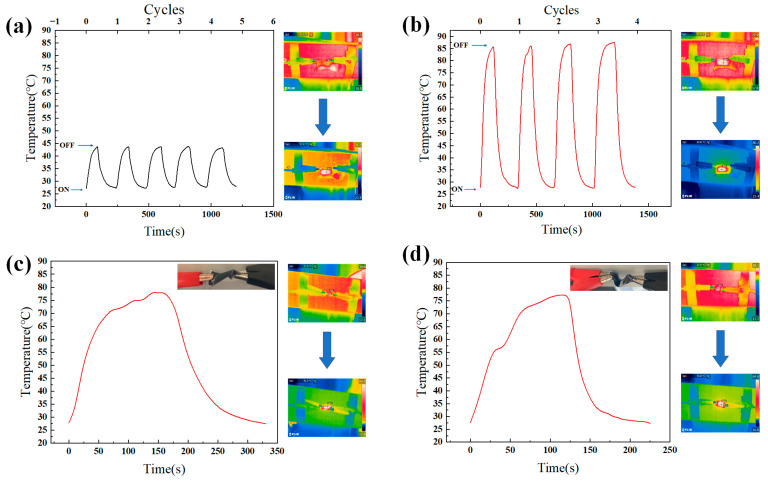
(**a**) Electrothermal temperature variation of the PDMS-CNTs-PVA composite film under 1 V. (**b**) Electrothermal temperature variation of the PDMS-CNTs-PVA composite film under 2 V. (**c**) Electrothermal temperature variation of twisted PDMS-CNTs-PVA composite film under 2 V. (**d**) Electrothermal temperature variation of bent PDMS-CNTs-PVA composite film under 2 V.

When the PDMS-CNTs-PVA films were energized at both ends with a voltage of 1 V, the films reached a maximum temperature of approximately 44 °C in about 90 s. After the power was removed, it took about 130 s for the films to return to room temperature. When the voltage was increased to 2 V, the films reached their maximum temperature of approximately 85 °C in 110 s, and it took approximately 200 s for the films to return to room temperature. In comparison, when a voltage of 1 V was applied to both ends of the CNT films, the maximum stable temperature was only 33 °C, while the PDMS-CNTs-PVA composite reached a maximum temperature of 44 °C. When a voltage of 2 V was applied, the maximum temperature of the PDMS-CNTs-PVA composite films was also higher than that of the CNT films. Therefore, compared to CNTs single films, the PDMS-CNTs-PVA composite films demonstrate superior energy-storage properties. In electrothermal experiments conducted by other researchers on CNT films [40,41], the temperature change rate of CNT films was consistently observed to be significantly faster than that of the composite films in this study. When both ends of the CNT film and the composite film were connected to a 2 V voltage, the composite film reached a maximum equilibrium temperature of 86.3 °C with a cooling rate of 0.295 °C/s, while the CNT film achieved a maximum equilibrium temperature of 45.5 °C with a cooling rate of 1.72 °C/s. The PDMS-CNTs-PVA composite film demonstrated superior energy storage and electrothermal properties compared to CNT films due to several key factors. The layered structure of the composite provides increased spatial capacity for energy storage and thermal management, while the PDMS network, comprising both crystalline and amorphous regions, enhances the film’s flexibility and structural stability. Additionally, the CNT layer within the composite acts as an air isolation barrier, mitigating environmental effects and reducing thermal losses. These combined attributes contribute to the enhanced performance of the composite film in energy-storage and electrothermal applications. This demonstrates that the PDMS-CNTs-PVA composite films have excellent energy storage properties, effectively controlling temperature diffusion, allowing the film’s temperature to rise gradually and maintain stability, and proving the superior electrothermal properties of the PDMS-CNTs-PVA composite films. (Figure 8a,b).

To verify the electrothermal properties of the material under different deformation conditions, the composite film was twisted 180° to create a twisted state, and bent 45° to create a bent state. Each sample was tested three times, and the results were averaged to obtain accurate experimental data (Figure 8c,d). Both types of film were energized at 2 V. The test results showed that the maximum temperatures of the bent and twisted films were both close to 80 °C, similar to that of the PDMS-CNTs-PVA composite films. This indicates that the composite films maintain stable and good electrothermal properties under different deformation states (Figure 8c,d). 

## 4. Conclusions

In this work, we presented an effective method for fabricating a conductive PDMS-CNTs-PVA composite film with excellent Joule heating and strain-sensing properties. Compared to existing single-layer materials, the sandwich composite structure demonstrated enhanced versatility, including superior elasticity, stretchability, electrothermal performance, and durability. The PDMS substrate leverages its intrinsic high elasticity and extensibility, allowing the composite to maintain structural integrity, even under significant deformation, making it ideal for flexible and stretchable device applications. The addition of PVA as the top layer imparts functional enhancements, particularly in wet-state sensitivity. The hydrophilic nature of PVA facilitates interactions with moisture, thereby enabling the composite to detect and respond to environmental moisture changes or direct liquid contact. The core of the composite, consisting of CNTs, provides exceptional electrical conductivity and the capability to reach temperatures as high as 108 °C under a relatively low voltage of 5 V. This combination makes the material highly suitable for applications in flexible heaters, thermal management systems, and other textile applications requiring precise temperature control and stability. During stretching, the composite films exhibited robust stability and sensing performance, with the resistance change rate increasing up to 25% as the degree of stretching escalated. Under compression, the composite film demonstrated a notable deformation response, with a resistance change rate of approximately 30%. In simulations of wet-state sensing, the composite film displayed distinct resistance changes, with resistance decreasing after water absorption. In electrothermal experiments, the CNT films exhibited rapid temperature changes, reaching up to 108 °C in 20 s at a voltage of 5 V with increasingly applied voltage. Moreover, the PDMS-CNTs-PVA composite film achieved higher temperatures at lower voltage levels, indicative of its efficient energy storage and superior electrothermal performance.

Therefore, this study provided a novel perspective for advancing research in multifunctional flexible sensors and electrothermal materials.

## Data Availability

This article includes all data.
